# Synchronous Presentation of Nodular Melanoma and Epithelioid Cell Melanoma: Case Report

**DOI:** 10.1155/crom/6396505

**Published:** 2025-07-15

**Authors:** Gianluca Gizzi, Dario Didona, Serge C. Thal, Luca Scarsella

**Affiliations:** ^1^Federazione Italiana Medici di Medicina Generale, Rome, Italy; ^2^Rare Diseases Center, Istituto Dermopatico dell'Immacolata, IDI-IRCCS, Rome, Italy; ^3^Department of Anesthesiology, Center for Clinical and Translational Research, Helios University Hospital Wuppertal, Wuppertal, Germany

**Keywords:** case report, epithelioid cell melanoma, ipilimumab, nivolumab, nodular melanoma, survival, synchronous presentation

## Abstract

**Introduction:** The synchronous occurrence of melanomas of varying histological types is an uncommon event, with reported incidences ranging from 0.2% to 8.6%.

**Case Report:** We present the case of a patient diagnosed with Stage IIB nodular melanoma and Stage IIIC epithelioid cell melanoma within a 3-month period. After surgical excision of both lesions, lymph node enlargement was observed in the obturator region, indicating metastatic spread. As a result, combined immunotherapy with nivolumab and ipilimumab was initiated. Nivolumab and ipilimumab were administered at doses of 1 and 3 mg/kg, respectively, every 3 weeks for a total of four doses. Thereafter, treatment was continued with nivolumab alone at a dose of 3 mg/kg every 2 weeks. The patient underwent three cycles of immunotherapy, initially combined with intravenous methylprednisolone, later transitioned to an oral regimen with dexamethasone. The patient initially demonstrated a favorable clinical response without adverse effects. However, after the third infusion, severe diarrhea developed, leading to daily fluid losses exceeding 8 L and associated hypokalemia. Therefore, methylprednisolone was administered intravenously (2 mg/kg/day). Additionally, the patient experienced a splenic infarction that resolved spontaneously without resulting in asplenia. At the most recent follow-up evaluation, no lymph node enlargement was detected, and surveillance continues at 3-month intervals.

**Discussion:** Although rare, the simultaneous emergence of melanomas at distinct anatomical sites underscores the necessity for increased patient vigilance and comprehensive clinical monitoring to facilitate early detection and timely intervention.

**Conclusion:** Prompt initiation of targeted immunotherapy may improve patient prognosis and outcomes.

## 1. Introduction

Invasive melanoma is the most lethal form of skin cancer, responsible for over 75% of skin cancer–related deaths [1]. The 5-year survival rate for metastatic cutaneous melanoma has been reported to be 39.4% [1]. Melanomas can present with various histopathological subtypes. Nodular melanoma (NM), which constitutes approximately 15%–30% of melanoma cases, is known for its rapid vertical growth phase and is associated with poorer clinical outcomes due to greater tumor thickness and aggressive biological behavior [2]. Epithelioid cell melanoma (ECM), characterized by a high degree of cellular dedifferentiation, is also linked to an unfavorable prognosis [3]. While surgical excision remains the standard treatment for localized melanoma, metastatic disease necessitates systemic therapy. Due to melanoma's resistance to conventional chemotherapy, advancements in genetically targeted treatments have significantly expanded therapeutic options for patients [4]. Nivolumab, a monoclonal antibody targeting the programmed cell death protein (PD)-1 receptor, enhances antitumor immunity by inhibiting a crucial negative regulator of T-cell activation. Ipilimumab, which blocks cytotoxic T-lymphocyte-associated protein 4 (CTLA-4), is another example of an immune checkpoint inhibitor utilized in systemic treatment.

**Figure 1:**
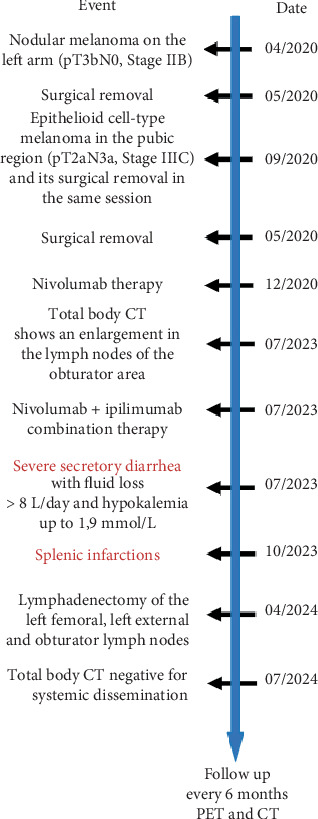
Timeline.

This report describes a case of synchronous NM and ECM, followed by adjuvant immunotherapy with nivolumab and ipilimumab due to systemic lymph node involvement. The patient experienced severe side effects, including significant secretory diarrhea and splenic infarction, leading to the premature cessation of therapy. However, the patient showed no evidence of systemic disease at the most recent follow-up, confirmed by total body computed tomography (CT) imaging.  Figure 1 illustrates the timeline of significant medical events from the initial melanoma diagnosis onward.

## 2. Case Report

In April 2019, during a routine screening of a pre-existing warty, raised, grayish lesion on the left arm, a new growth measuring 0.5 cm in diameter was identified. The patient, with no significant medical history (detailed in Table 1), reported continuous minor bleeding over the past weeks, prompting further evaluation due to its suspicious nature.

In May 2020, a skin excision measuring 3 × 1.5 × 1 cm, encompassing the lesion and surrounding tissue, was performed. Histopathological analysis confirmed a diagnosis of NM with the following features: Breslow thickness of 2.1 mm, ulceration with a linear extension of 1.8 mm, a mitotic index of 10/mm^2^, no microsatellites, clear linear and deep margins, nonbrisk tumor-infiltrating lymphocytes (TILs), Clark Level IV, absence of regression, and no lymphovascular or perineural invasion. No associated melanocytic nevus was present. Solar dermal elastosis was graded as Grade 2, according to the World Health Organization (WHO) classification [5]. Genetic analysis identified mutations in the neuroblastoma RAS (NRAS) (Q61L) gene and a V-raf murine sarcoma viral oncogene homolog B (BRAF) wild-type status. A total body CT scan conducted in June 2020 showed no evidence of systemic metastasis.

In July 2020, the patient underwent a wide local excision and a left axillary sentinel lymph node biopsy. Both the surrounding skin and the sentinel lymph node were free of metastatic disease. The case was staged as Stage IIB (pT3b, N0, and M0) based on the American Joint Committee on Cancer (AJCC) guidelines [6].

In September 2020, a follow-up visit revealed a new suspicious lesion in the pubic region, which was excised. Histopathology confirmed an ECM with a Breslow thickness of 1.9 mm, no ulceration, and a mitotic rate of 3/mm^2^. Genetic analysis identified a BRAF V600E mutation and NRAS wild-type status. In October 2020, the patient underwent a wide excision of the lesion (6 × 2 × 3 cm) and a bilateral sentinel lymph node biopsy, involving left and right inguinal lymph nodes. Histological analysis revealed multifocal metastases in four out of seven left inguinal lymph nodes, with the largest focus measuring 0.3 mm and no subcapsular extension. One right inguinal lymph node also showed multifocal metastases without extracapsular extension, with the largest focus measuring 0.07 mm. The ECM was thus classified as Stage IIIC (pT2a, N3a, and M0) according to AJCC 8^th^ edition. A multidisciplinary team reviewed the case, and, given the bilateral nature of the lesions, the decision was made in agreement with the patient to avoid deep bilateral inguinal lymphadenectomy.

Adjuvant immunotherapy with nivolumab (1 mg/kg) was initiated in December 2020. The treatment was well tolerated for 12 months, with no reported adverse effects. Follow-up total body CT scans showed no progression or new metastases, and blood tests remained within normal ranges. However, in July 2023, a follow-up CT scan detected lymph node enlargement in the left obturator region, measuring 22 mm in the short axis. In August 2023, an 18F-fluorodeoxyglucose (18F-FDG) positron emission tomography (PET) scan revealed hyperfixation in the obturator lymph nodes, with a maximum standardized uptake value (SUV) of 8 and dimensions of 35 × 22 mm, suggestive of recurrence. This was not further evaluated by biopsy. No other areas of abnormal 18F-FDG uptake were observed in the lungs, mediastinum, liver, spleen, pancreas, adrenal glands, or bones Stage IIIa (pT2a, N1, and M0) according to AJCC 8^th^ edition.

A combined therapy of nivolumab and ipilimumab was initiated in July 2023 as first-line treatment in a metastatic setting, administered every 21 days. A total of three chemotherapy cycles were administered. Initially, therapy with methylprednisolone at a dose of 60 mg every 12 h was associated; subsequently, the treatment was converted to an oral regimen with 125 mg/day. Although the first two infusions were well tolerated, 8 h after the third infusion, the patient developed severe nausea, diffuse abdominal pain, and nonbloody diarrhea classified as Grade 4 [7]. The condition worsened, with fluid loss reaching up to 10 L/day and associated vomiting, necessitating hospitalization in September 2023. The patient lost 7 kg within 1 week of symptom onset. Blood tests revealed severe hypokalemia (potassium level of 1.9 mEq/L) with normonatremia (sodium level of 140 mEq/L). Infectious causes were excluded, and treatment was initiated with methylprednisolone (60 mg every 12 h), octreotide (0.3 mg by continuous subcutaneous infusion every 48 h), and levosulpiride (25 mg every 12 h). Clinical improvements in blood parameters and hydration status allowed discharge in October 2023, with Grade 1 diarrhea persisting.

**Figure 2:**
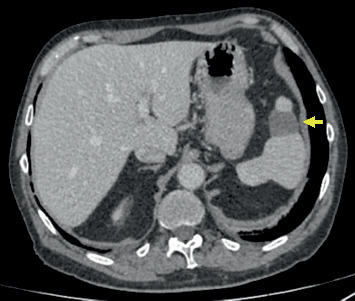
Computed tomography (CT) scan of the abdomen showing a splenic infarction. The yellow arrow indicates the infarcted area in the anterior region of the spleen.

Six hours postdischarge, the patient was readmitted due to sudden-onset abdominal pain localized to the left upper quadrant. An abdominal CT scan revealed multiple splenic infarctions. Revaluation 17 days later confirmed stable findings, as shown in Figure 2.

After 2 days of inpatient observation, the patient was discharged with a home therapy plan, continuing octreotide for 7 days and tapering prednisone, which was discontinued in December 2023. Grade 1 diarrhea persisted until December 2023 but resolved completely. Due to these adverse events, immunotherapy was discontinued, and palliative lymphadenectomy of the left femoral, left external, and obturator lymph nodes was performed in April 2024. Histopathological examination confirmed that all excised lymph nodes and paranodal adipose tissue were free of metastatic disease.

A follow-up total body CT scan in July 2024 showed no evidence of systemic recurrence. Continued surveillance includes biannual total body CT and PET scans.

## 3. Discussion

We present a clinical case of a patient with two melanomas of distinct histological types within a 6-month period, located in different anatomical regions. Notably, there was no evidence of significant risk factors such as a family history of melanoma, genetic disorders, prior melanoma diagnoses, or an immunocompromised state. The patient also denied prolonged or intense sun exposure. The exact timeline of melanoma onset could not be determined, as the patient had not previously undergone dermatological evaluations.

Severe diarrheal episodes, including Grade 4 diarrhea, during immunotherapy with combined nivolumab and ipilimumab have been reported in the literature, though this level of severity remains a rare occurrence [8]. Several studies have demonstrated a positive correlation between the occurrence of severe adverse events during immunotherapy and improved clinical outcomes in patients undergoing immune checkpoint inhibitor therapy. Patients who develop high-grade (Grades 3 and 4) adverse events, commonly referred to as immune-related adverse events (irAEs), often exhibit enhanced therapeutic responses, including higher objective response rates and prolonged progression-free and overall survival [9, 10]. This phenomenon is thought to reflect a more robust activation of the immune system, which, while contributing to toxicity, may also result in more effective antitumor activity [11]. A multidisciplinary approach is essential for the effective management of irAEs associated with immunotherapy. Involving oncologists, organ-specific specialists, pharmacists, and nurses enables early detection, accurate diagnosis, and timely intervention, thereby improving patient outcomes. Clinical guidelines from societies such as the American Society of Clinical Oncology, the National Comprehensive Cancer Network, and the Society for Immunotherapy of Cancer endorse this model, and its successful implementation in academic centers has demonstrated reduced toxicity-related complications and more coordinated care [12–14].

Spleen infarctions are extremely rare as side effects of the combined therapy with nivolumab and ipilimumab, with only two clinical cases of autosplenectomy having been described so far in the literature [15, 16]. This underscores the rarity of the observed complications in this case, highlighting the importance of clinical vigilance and prompt management of severe side effects associated with immunotherapy.

## 4. Conclusion

Further research is warranted to understand the mechanisms behind such rare adverse events and to develop strategies for early identification and intervention. The management of immune-related toxicities remains a critical aspect of patient care to optimize treatment outcomes while minimizing risk.

## Figures and Tables

**Table 1:** Patient characteristics.

**Patient characteristics**	
Age	67 years
Sex	Male
Body mass index (BMI)	23.9
Skin phototype according to Fitzpatrick	III
Family history of melanoma	None
Medical history	Bilateral knee osteoarthritis; right knee arthroplasty
Chronic medications	None at the time of diagnosis

## Data Availability

Data sharing is not applicable to this article as no new data were created or analyzed in this study.
